# Risk prediction in intrahepatic cholangiocarcinoma: Direct comparison of the MEGNA score and the 8^th^ edition of the UICC/AJCC Cancer staging system

**DOI:** 10.1371/journal.pone.0228501

**Published:** 2020-02-03

**Authors:** Felix Hahn, Lukas Müller, Aline Mähringer-Kunz, Sebastian Schotten, Christoph Düber, Jan B. Hinrichs, Sabine K. Maschke, Peter R. Galle, Fabian Bartsch, Hauke Lang, Arndt Weinmann, Roman Kloeckner

**Affiliations:** 1 Department of Diagnostic and Interventional Radiology, Johannes Gutenberg-University Medical Center Mainz, Mainz, Germany; 2 Department of Diagnostic and Interventional Radiology, Hannover Medical School, Hannover, Germany; 3 Department of Internal Medicine, Johannes Gutenberg-University Medical Center Mainz, Mainz, Germany; 4 Department of General, Visceral and Transplant Surgery, Johannes Gutenberg-University Medical Center Mainz, Mainz, Germany; Universitatsklinikum Hamburg-Eppendorf, GERMANY

## Abstract

**Background:**

External validation of prognostic risk models is essential before they are implemented in clinical practice. This study evaluated the recently developed MEGNA score for survival prediction after resection of intrahepatic cholangiocarcinoma (ICC), with a focus on the direct comparison of its prognostic value to that of the current International Union Against Cancer (UICC)/American Joint Committee on Cancer (AJCC) Cancer staging system.

**Material and methods:**

Between 1997 and 2018, 417 consecutive patients with ICC were referred to our tertiary care centre and were retrospectively identified out of a dedicated clinical database. Of this group, 203 patients underwent surgical resection and met the inclusion criteria. Multivariate analysis was performed to assess the predictors of the recently proposed MEGNA score regarding overall survival (OS). Concordance indices (C-indices) and integrated Brier scores (IBS) were calculated to assess the ability of both the MEGNA score and the current (8^th^) edition of the UICC/AJCC Cancer staging system to predict individual patient outcome.

**Results:**

Stratification according to the MEGNA score resulted in a median OS of 34.5 months, 26.1 months, 21.5 months, and 16.6 months for MEGNA scores 0, 1, 2, and ≥3, respectively (log rank p < 0.001). However, of the five factors that contribute to the MEGNA score, age > 60 years was not a predictor for poor OS in our cohort. The C-index for the MEGNA score was 0.58, the IBS was 0.193. The 8^th^ edition of the UICC/AJCC system performed slightly better, with a C-index of 0.61 and an IBS of 0.186.

**Conclusion:**

The ability of the MEGNA score to predict individual patient outcome was only moderate in this external validation. Its prognostic value did not reach that of the more widely known and used UICC/AJCC system. However, neither scoring system performed well enough to support clear-cut clinical decisions.

## Introduction

Intrahepatic cholangiocarcinoma (ICC) is the second most common primary liver malignancy after hepatocellular carcinoma (HCC). Prevalence is highest in Asia, but incidence in the low endemic western countries is estimated at approximately 0.4–2.0/100,000, and has reportedly increased during the last three decades [1–4].

Affected patients are often asymptomatic in the early stages, and at diagnosis the tumour is often already at an advanced stage [5]. Tumour recurrence is reported in about 65% of patients [6].

The TNM system of the International Union Against Cancer (UICC)/American Joint Committee on Cancer (AJCC) is widely used for ICC staging. However, staging was identical for HCC and ICC until the 6^th^ edition of this staging system; differences between these two distinct tumour types were introduced in the 7^th^ edition of the staging system and updated in its current (8^th^) edition [7].

Other scores and nomograms have been developed and evaluated: the Fudan score stratifies risk groups depending on serum alkaline phosphatase level, carbohydrate antigen 19–9 (CA19-9) level, the number and diameter of intrahepatic tumour(s), and the tumour boundary type as determined upon cross-sectional imaging [8]. The Hyder nomogram depends on age, tumour size, multifocality, nodal status, vascular invasion, and presence/absence of cirrhosis [9]. An external evaluation of these scoring systems with 188 patients showed an overall survival (OS) prediction accuracy according to concordance index calculation of 0.55 with the Fudan score and 0.66 using the Hyder nomogram [10]. Hence, tenctheir predictive power was considerably lower in that evaluation than in the original publications.

In 2017, Raoof et al. developed a novel scoring system to predict outcomes of patients with ICC after resection [11]. Their MEGNA score consists of five factors: age > 60 years, multifocality, extrahepatic tumour extension, tumour grading, and lymph node metastasis. Extrahepatic tumour extension was defined as “any perforation of the visceral peritoneum, invasion of the hepatic artery or vena cava, and involvement of surrounding viscera”. The authors themselves validated their score using an independent data set from the National Cancer Institute’s Surveillance, Epidemiology, and End Results (SEER) registry, and came to the conclusion that MEGNA is superior in predicting patient survival after hepatectomy compared with current staging systems [11].

Recently, Schnitzbauer et al. performed a multicentre validation of the MEGNA score, and came to the conclusion that the risk groups as proposed by the MEGNA score result in a significant stratification regarding OS [12]. However, a direct comparison with the current UICC cancer staging system was not presented. Therefore, to the best of our knowledge, the claim of the original MEGNA authors has not been confirmed to date.

## Materials and methods

This study was approved by the responsible ethics committee (Ethics committee of the Medical Association of Rhineland Palatinate, Mainz, Germany) for the retrospective analysis of clinical data (permit number 2018–13618) and the requirement for informed consent was waived. Additional examinations were not performed. Patient records and information were anonymized and de-identified prior to analysis.

**Fig 1:**
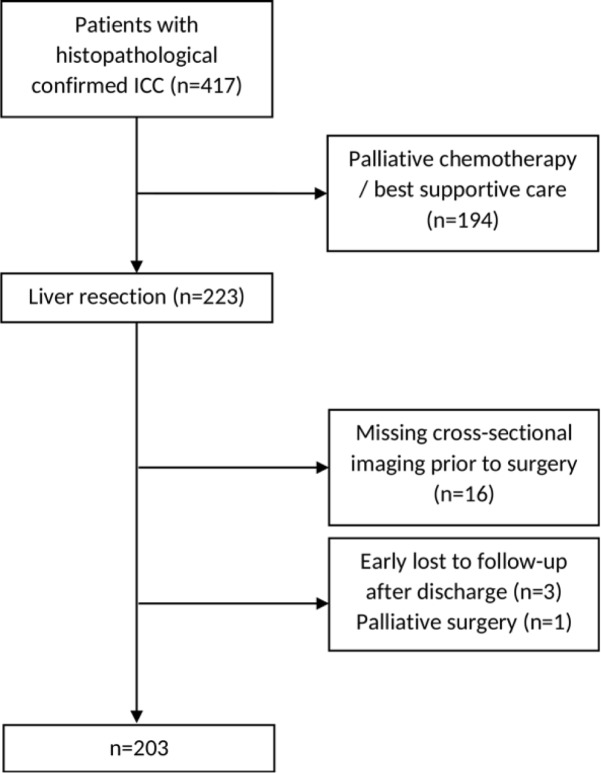
CONSORT flow chart. The reasons for drop-outs and the final number of patients for whom the MEGNA score could be evaluated are shown.

Between January 1997 and January 2018, 417 patients with histologically confirmed ICC were referred to our tertiary care centre and were retrospectively identified out of an established clinical registry software for the characterization of patients with HCC and ICC [13]. Follow-up ended February 2019. Of these patients, 223 underwent liver resection. An additional 20 patients were excluded as described in Fig 1; the remaining 203 patients were included in the final analysis. Contrast-enhanced computed tomography or magnetic resonance imaging prior to surgery was evaluated regarding tumour size and number of intrahepatic lesions, as well as presence of distant metastases.

Histopathology reports were extracted from the hospital information system, and follow-up visits were extracted from the hospital and radiology information systems. Death dates were enquired at the appropriate Resident’s Registration Offices.

Statistical analysis was performed using R 3.5.1 [14]. The the packages “survival” and “survminer” (https://cran.r-project.org/package=survival, https://cran.r-project.org/package=survminer, accessed 31.01.2019) were used for survival analysis, “Hmisc” (https://cran.r-project.org/package=Hmisc, accessed 31.01.2019) for computation of Harrell’s C concordance-indices, and “pec” for computation of prediction error curves (https://cran.r-project.org/package=pec, accessed 31.01.2019).

Binary and categorical data are reported as absolute numbers and percentages. All continuous data are presented as mean ± standard deviation. A p-value of <0.05 was considered statistically significant. Log-rank tests and Kaplan Meier curves were used for survival analysis between strata. Multivariate Cox proportional hazards regression models were fitted to determine the influence of predictors. Validation was performed using Harrell’s concordance index (C-index) [15]. The C-index provides the probability a randomly selected patient who experienced an event (in our case death) had a higher risk score than a patient who had not experienced the event: “A value of c of .5 indicates random predictions, and a value of 1 indicates perfect prediction (…). A model having c greater than roughly .8 has some utility in predicting the responses of individual subjects.” [16]. Prediction error curves were based on the Brier score. For a single subject, the Brier score at time t is defined as the squared difference between observed survival status (e.g., 1 = alive at time t and 0 = dead at time t) and the predicted outcome probability [17]. The integrated Brier score (IBS) over the interval [0 months, 60 months] was calculated as a summary measure of prediction error.

## Results

In our cohort, the mean patient age at diagnosis was 62.3 years (median 63.3 years). Median follow-up for all patients was 21.7 months, a total of 30 patients were lost to follow-up. At the end of the study period, 166 deaths had occurred in the entire cohort. Detailed baseline characteristics of all patients are provided in Table 1.

**Table 1:** Baseline characteristics of patients with ICC in this study.

Age, years	Mean ± SD; IQR	62.3 ± 11.7; 54.9–70.6
**Sex, n (%)**	Male	104 (51.2)
	Female	99 (48.8)
**Number of intrahepatic lesions, n (%)**	1	159 (78.3)
	2	18 (8.9)
	3	7 (3.4)
	4	4 (2.0)
	≥5	15 (7.4)
**Tumour size, mm**	Mean ± SD; IQR	82 ± 45; 45–110
UICC T stage, n (%)*	T1a	45 (22.2)
	T1b	48 (23.6)
	T2	59 (29.1)
	T3	32 (15.7)
	T4	19 (9.4)
**Extent of surgery, n (%)**	Minor	54 (26.6)
	Major	149 (73.4)
**Surgical resection margin, n (%)**	R0	171 (84.2)
	R1	30 (14.8)
	R2	2 (1.0)
**Lymphadenectomy, n (%)**	not performed	59 (29.1)
	≤5 lymph nodes	86 (42.3)
	≥6 lymph nodes	58 (28.6)
**Nodal status, n (%)**	Negative	167 (82.3)
	Positive	36 (17.7)
**Grading, n (%)**	Low-intermediate	137 (67.5)
	High	66 (32.5)
**Initial subsequent treatment in case of recurrence, n (%)**	Re-Resection / Surgery	14 (6.9)
	Locoregional therapy	25 (12.3)
	Systemic chemotherapy	83 (40.9)
	Best supportive care	15 (7.4)

Abbreviations: SD = standard deviation; IQR = interquartile range; UICC = International Union Against Cancer.

*According to the current 8^th^ edition.

The MEGNA score weighs the following factors with one point each: age > 60 years, multifocality, extrahepatic extension, high tumour grading, and node positivity. In the original MEGNA publication, four risk groups were stratified depending on the MEGNA score: 0 low risk, 1 intermediate risk, 2 high risk, and 3–5 very high risk.

In uni- and multivariate Cox proportional hazard analyses, all factors with the exception of age > 60 years were associated with an increased hazard ratio (Table 2).

**Table 2:** Uni- and multivariate Cox proportional hazards regression model evaluating the MEGNA factors.

Factor	Univariate analysis			Multivariate analysis		
	HR	95% CI	p-value	HR	95% CI	p-value
Age > 60 years	0.89	0.65–1.23	0.493	0.88	0.64–1.21	0.427
Nodal status	1.82	1.24–2.68	0.002	1.52	1.02–2.26	0.041
Multifocality	1.62	1.13–2.32	0.008	1.74	1.21–2.51	0.003
Extrahepatic extension	2.17	1.33–3.53	0.002	2.04	1.24–3.35	0.005
Grading	1.76	1.27–2.43	0.001	1.74	1.25–2.42	0.001

Abbreviations: HR = hazard ratio, CI = confidence interval.

**Fig 2:**
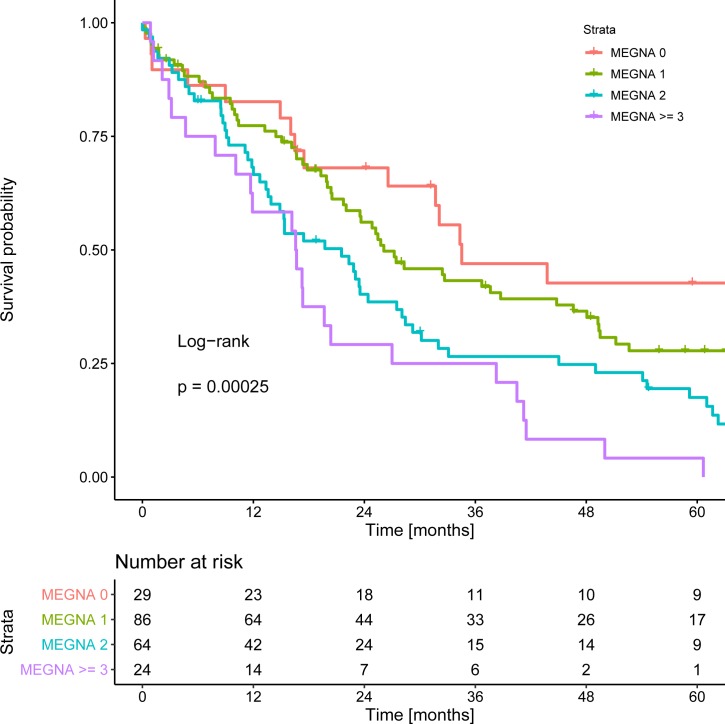
Kaplan Meier curves of overall survival stratified according to MEGNA.

Of the 203 patients, 29 patients were categorized as MEGNA low risk, 86 were categorized as intermediate risk, 64 were categorized as high risk, and 24 were categorized as very high risk. Median OS for each risk group was 34.5 months, 26.1 months, 21.5 months, and 16.6 months, respectively (Log rank test p-value < 0.001; Fig 2). Concordance index computation yielded a Harrell’s C-index of 0.58.

**Fig 3:**
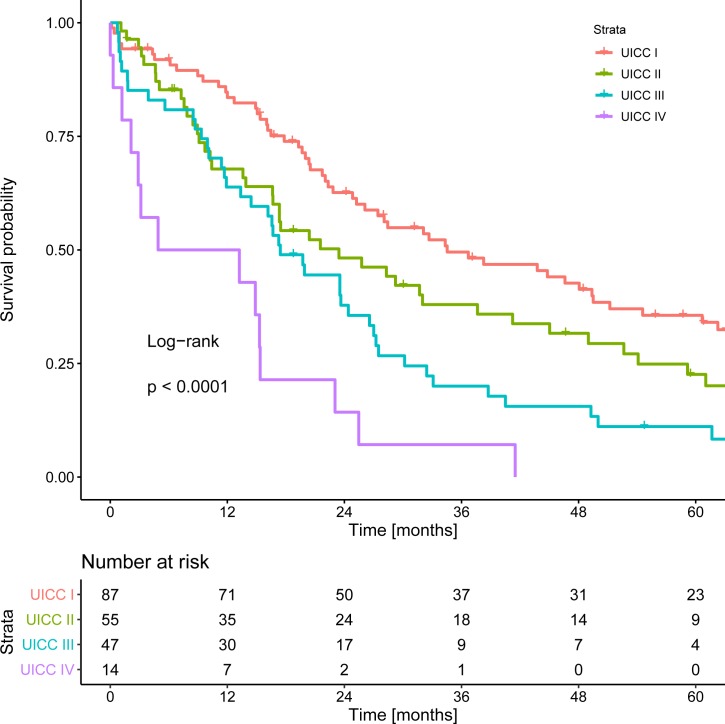
Kaplan Meier curves of overall survival stratified according to UICC.

When grouped according to the latest UICC classification (8^th^ edition), 87 patients were classified as stage UICC I, 55 as UICC II, 47 as UICC III, and 14 as UICC IV. Median OS for each stage was 34.5 months, 23.4 months, 17.4 months, and 9.1 months, respectively (Log rank test p-value < 0.001; Fig 3). Concordance index computation yielded a Harrell’s C-index of 0.61.

**Fig 4:**
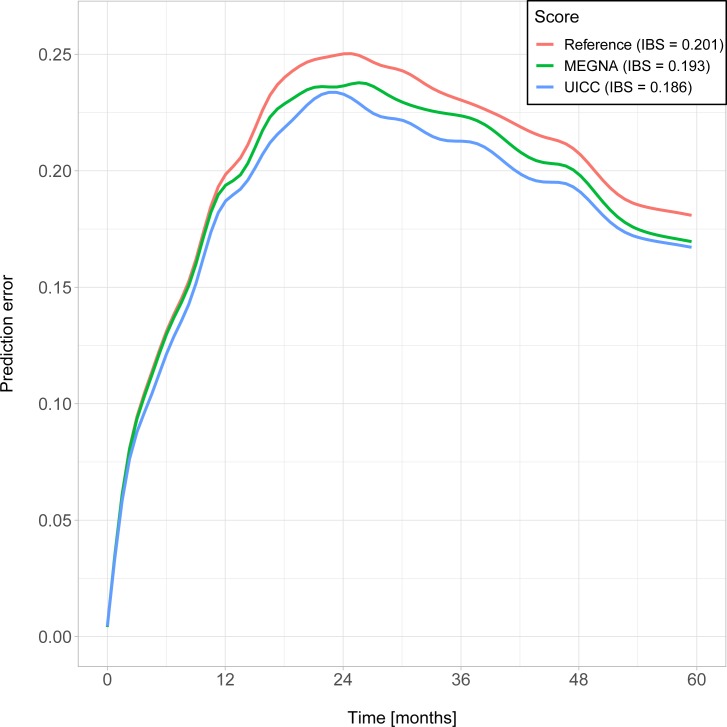
Smoothed prediction error curves and integrated Brier Scores (IBS) for Kaplan Meier estimates based on the MEGNA and UICC stratification as well as on estimates for all patients without any stratification (Reference curve).

Prediction error curves based on the Brier score are shown in Fig 4. The IBS over the interval [0 months, 60 months] was 0.193 when using the MEGNA score and 0.186 when using the UICC stages. In comparison, the IBS was 0.201 using the Kaplan-Meier estimates for the unstratified sample.

## Discussion

In this study, the MEGNA score was able to differentiate median OS after ICC resection between the risk groups. However, due to overlap in the survival time distributions, concordance index calculation yielded a value of only 0.58.

The suggested age cut-off of 60 years was not associated with an increased hazard ratio in our cohort, thus rendering one factor out of five entirely dispensable and adding one degree of uncertainty to the risk stratification. Whether age is an additional independent predictor of OS is unclear in the literature. It is considered a risk factor in the MEGNA score and the Hyder nomogram [9,11]; however, it is not included in the Fudan score or the Wang nomogram [8,18]. Without the age component, an abridged MEGNA score consisting of the remaining four factors and the same stratification into four risk groups–a MEGN score, so to speak–led to a slightly better Harrell’s C-index of 0.61.

Regarding differences between the original study population in the paper by Raoof et al. and our cohort, tumour size was larger in our cohort than in the original (median 7.4 cm, IQR 4.5–11.0 cm vs median 5.5 cm, IQR 3.5–8.0 cm) [11]. Node positivity was slightly less compared with the original cohort (18% vs 20%). Age (median 63 y, IQR 55–71 y vs 65 y, IQR 55-72y), multifocality (unifocal 78% vs 81%), extrahepatic extension (9% vs 10%), and grading (poorly differentiated 33% vs 30%) were comparable between the two cohorts [11]. Thus, the cohorts were quite similar regarding the MEGNA factors; given this level of similarity a better performance of the MEGNA score was expected a priori.

Regarding lymphadenectomy, it is important to note that the share of patients who underwent lymph node resection in our cohort is on the low end of published data [19]. The reason for this lies primarily in the long period of patient recruitment, and lymphadenectomy was not routinely performed in the early years. However, the number of portal lymphadenectomies in the original cohort by Raoof et al. was low as well (45%), and the share of patients with lymph node metastases was not statistically different between our and the original cohort (36/203 vs 52/262, p = 0.63).

Very recently, Schnitzbauer et al. performed a multicentre analysis of the MEGNA score and validated the score in their study. They described an overall survival at the end of the 5 year follow-up period of 68%, 48%, 32%, and 19%, respectively, for MEGNA groups 0, 1, 2, and 3 [12]. Thus, they presented higher survival rates than in our cohort (43%, 28%, 18%, and 4%). This is likely attributable to our long recruitment period as well since both initial surgical approaches and subsequent therapies including systemic chemotherapy have improved towards the second half of the study period [19,20]. Of note, age > 60 years was not a significant factor towards OS in the study by Schnitzbauer as well. Interestingly, high tumour grading was not a significant factor in their cohort. A multivariate analysis of the MEGNA factors is not presented in their study. Moreover, the authors do not present a direct comparison of the MEGNA score to the UICC/AJCC system.

In our cohort, and in contrast to the original publication, performance of the MEGNA score was inferior to the more widely known and used UICC/AJCC system. The results yielded by UICC stratification were marginally better, with a C-index of 0.61. When stratified according to the UICC stages, patients in UICC stages II and III in particular showed overlap of survival curves, supporting the predictive value of intrahepatic vascular invasion and intrahepatic metastasis [8,21]. However, concordance indices in the vicinity of 0.6 must be considered as only moderate, which applies to both the MEGNA score and the UICC system [16].

Because risk scores for liver malignancies and in general are developed on a particular data set and are prone to a certain degree of overfitting, there are many instances of scores and survival predictors that have a history of mediocre reproducibility in external validations, for both ICC and HCC [10,22,23].

Moreover, a limitation most prediction scores face is that they try to predict survival at a very early time point, usually at initial diagnosis or immediately post-resection. Therefore, relevant following treatments like locoregional or systemic therapies that happen later in the patients’ course of disease cannot be taken into account, even though their influence on survival has been demonstrated [20,24].

With surgical resection being the only potential cure for affected patients, the decision is often made to resect large ICCs or tumours with extrahepatic extension. Bergeat et al. reported no negative effect of oncologic outcomes in extended liver resections, but noted an increased risk of major complications [25]. Spolverato et al. came to the conclusion that liver resection can be performed in patients with large or multifocal ICC with similar postoperative complications, but observed a worse 5-year OS [26]. In case of ICC recurrence, the safety of repeat hepatic resection has been demonstrated, with long-term survival outcomes [27].

Therefore, even though stratification according to both the MEGNA risk score and the UICC staging system resulted in significant log rank p-values, due to the number of deaths across all groups, the discriminative ability of the scores to predict survival for a randomly selected patient out of one group was only moderate. That is why in clinical practice, as surgical resection is the only curative treatment, patients will be operated on if oncologically reasonable, regardless of the MEGNA score.

Our analysis has several limitations. First, the study was conducted at a single centre. Second, the validation was conducted retrospectively and the final sample size (n = 203) was only moderate. Due to the long study period, improved treatment options carry a bias that is difficult to control for: as there was a lack of systematic lymphadenectomy in the early years, a bias due to missed positive lymph nodes has to be assumed. Moreover, patients received different chemotherapy regimen over time; the current standard of gemcitabine and cisplatin was introduced in 2010 following the UK-ABC2 trial and outcomes might have improved towards the end of the recruitment period due to adherence to this regimen [20]. We actively decided against imputing missing values and included only patients for whom all data needed for the analysed scores was available, and therefore reduced patient numbers and statistical power in favour of data completeness.

## Conclusion

The predictive ability of the MEGNA scoring system was only moderate in this external validation, and its prognostic value did not reach that of the more widely known and used UICC/AJCC system. Because neither of the investigated scoring systems performed well enough to support clear-cut clinical decisions, interdisciplinary tumour boards and individual approaches are necessary to determine the appropriate course of action for patients with advanced ICC.
